# Male and Female Users’ Differences in Online Technology Community Based on Text Mining

**DOI:** 10.3389/fpsyg.2020.00806

**Published:** 2020-05-26

**Authors:** Bing Sun, Hongying Mao, Chengshun Yin

**Affiliations:** School of Economics and Management, Harbin Engineering University, Harbin, China

**Keywords:** online technology community, gender differences, Latent Dirichlet Allocation (LDA) topic model, machine learning, regression analysis

## Abstract

With the emergence of online communities, more and more people are participating in online technology communities to meet personalized learning needs. This study aims to investigate whether and how male and female users behave differently in online technology communities. Using text data from the Python Technology Community, through the LDA (Latent Dirichlet Allocation) model, sentiment analysis, and regression analysis, this paper reveals the different topics of male and female users in the online technology community, their sentimental tendencies and activity under different topics, and their correlation and mutual influence. The results show the following: (1) Male users tend to provide information help, while female users prefer to participate in the topic of making friends and advertising. (2) When communicating in the technology community, male and female users mostly express positive emotions, but female users express positive emotions more frequently. (3) Different emotional tendencies of male and female users under different topics have different effects on their activity in the community. The activity of female users is more susceptible to emotional orientation.

## Introduction

The rapid development of modern information technology has affected people’s learning, work, and lifestyle. It is urgent for societal members to make full use of information technology to explore the digital learning mode and meet the growing diversified and personalized learning needs (Johnson and Ambrose, 2006). The Internet provides an increasingly popular platform for the public; users with common interests or goals can exchange information, express opinions, seek emotional support, and establish social relations with others (Lu Y. J. et al., 2013; Park and Park, 2014).

An online technology community can aggregate distributed information and knowledge; its existence meets people’s personalized demand for knowledge and social demand for interpersonal relationships (Chen et al., 2019). Understanding the gender differences of users’ topics and emotions in the online technology community can help broaden the understanding of community users, so as to better serve users in a targeted manner, which will help promote the sustainable development of online technology communities.

Gathering people with similar interests into a group and studying the behavioral differences among different groups is helpful to have a deeper understanding of the interests of each group and provide guidance for understanding the information needs, emotional needs, and relationship needs of different groups (Liu et al., 2018). The WHO defines gender in this way: “Gender refers to the socially constructed characteristics of women and men—such as norms, roles and relationships of and between groups of women and men. It varies from society to society and can be changed.” In undertaking a review of a major individual difference variable such as gender, it is helpful to identify some underlying principles that can generate predictions and unite an otherwise disparate collection of individual findings (Loftus et al., 1987). User segmentation by gender is an efficient and audience-oriented viable segmentation method to differentiate markets and services (Kraft and Weber, 2012). The theories of gender differences mainly include social–cultural theory (Wood and Eagly, 2012), anthropology evolution theory (Tooby and Cosmides, 2005), and selective hypothesis (Meyers-Levy, 1989; Meyers-Levy and Maheswaran, 1991; Meyers-Levy and Sternthal, 1991). These theories are more complementary, and most of the findings of gender differences can be explained by one or more of the above viewpoints (Meyers-Levy and Loken, 2015). In addition, all gender research methods now recognize socio-cultural factors (such as social and cultural role learning, stereotypes, media, and marketing information). These theories lay the foundation for the study of gender differences.

A traditional limitation of gender difference research is the large sample size required to reach broad conclusions. Today, the emergence of user-generated content solves this problem because the Internet can provide many texts on various topics (Teso et al., 2018). The second limitation concerns the methodological perspective in the analysis of gender differences. When the data sample is large, it is difficult to carry out qualitative analysis. On the contrary, text mining is an effective solution (Liu et al., 2018; Teso et al., 2018).

In recent years, online community posting text has been used by some scholars to study the use behavior of community users, the role of knowledge dissemination, and other issues (Chau and Xu, 2012; Teso et al., 2018). An online technology community is a reliable way to produce high-quality and cost-effective software and technology (Mockus et al., 2000). Although scholars have studied the user role and continuous contribution behavior of the online technology community (Singh and Holt, 2013; Cai and Zhu, 2016), they have ignored the research on male and female users’ differences in the online technology community, and considering gender differences may improve the understanding of the relationship between community user participation and their sentiments, which needs further research. Therefore, this paper uses an online technology community with the characteristics of the times and representativeness and adopts text mining methods to overcome the above two limitations, to analyze the subject needs and sentimental differences of user communication in the online technology community for different genders, as well as the relationship between user activity and sentiment. It will help community managers to effectively meet and guide the needs of community users and thus contribute to the sustainable development of online technology communities.

## Literature Review

### Research on Gender Differences

#### Gender Differences in General

For a long time, the difference of language use and emotion between men and women has been the interest of gender difference research. Previous studies on gender differences in language use have shown that men are more likely to use language to convey information, while women are more likely to use language to communicate for social purposes (Newman et al., 2008). Women are more likely to ask questions in daily conversations, while men are more direct and use more commands to tell people to do something. The study also found that women also use longer sentences, while men generally use more words and have more opportunities to speak in conversation (Mulac and Lundell, 1994). Regarding emotional research, most studies report that women refer to emotions more often than men (Thomson and Murachver, 2001).

#### Gender Differences in Computer-Mediated Communication

The emergence of the Internet and Web2.0 provides new opportunities for the study of gender differences. The scale and number of computer-mediated support groups have been increasing; their relatively unique feature is the lack of social and physical cues, which gives participants the opportunity to remain fully anonymous (Davison et al., 2000). However, can the unique characteristics of computer-mediated support groups eliminate some gender differences in face-to-face communication and support seeking? Some scholars have challenged the lack of gender differences in computer-mediated support groups. Some studies have shown that patient support group participants are more likely to be female; for instance, within the cancer literature, women have been found to outnumber men at a rate of four to one in many support groups (Krizek et al., 1999). Bellman et al. (1993) found that women in an anonymous online bulletin board were more likely to adopt an aggressive and assertive manner online than in face-to-face interactions. In an analysis of 3,000 news support group postings, Witmer and Katzman (1997) found that women have more posts and challenges in their posts than men. Wolf (2000) found that while females show a strong preference for support groups that focus on the discussion of emotional issues, males appear to prefer information-oriented support groups.

#### Gender Differences in Online Communities

The emergence of user-generated content and online communities has broadened the scope of gender research (Zhang et al., 2013). Teso et al. (2018) analyzed the shared comments of an online e-book brand community from the perspective of gender, pointing out that women like lifestyle books, while men like science fiction and humor books. Liu et al. (2018) analyzed the post texts of an online medical community, and the results showed that male users’ post information is usually more professional, and female users are more inclined to seek emotional support in a healthy community. At the same time, female users showed more negative emotions.

One of the reasons why the research on gender differences of computer-mediated online support groups has contradictory conclusions may be that the sampling strategies of the research are quite different, that is, the number of messages and the time period of analysis (Mo et al., 2009). Using all the text data of an online community can overcome the obstacles of a small number of messages and a specific time period, and can provide a broader conclusion for the study of gender differences. The online technology community provides a new platform for people to acquire knowledge and information and enables some knowledge and information stored in the human brain but not found by search engines to be reflected, and the interactive way of posting and replying has promoted the community users’ knowledge exchange (Chen et al., 2019). The online technology community can provide all text content in all time periods, so it can provide valuable and comprehensive insights for users’ online learning. In particular, the positive impact of interaction in the open source software community of the online technology community has been the opportunity to learn in the process of participation (Koh et al., 2007). However, only some scholars have studied the user behavior of the open source community (Barcellini et al., 2008; Cai and Zhu, 2016), ignoring the research on gender differences. It is necessary to study gender differences in the specific social context of the online technology community to understand how individuals use the online technology community. Therefore, this paper will study the gender differences by analyzing users’ posting texts in the online technology community, so as to expand the existing research horizons and technical methods.

### Text Mining

In 1995, Feldman first proposed the concept of text mining; it is a process of discovering and extracting hidden and previously unknown knowledge from a large amount of text data by combining machine learning and information retrieval technology, and ultimately forming valuable knowledge (Forman et al., 2008). Text mining mainly includes two parts: topic modeling and sentiment analysis.

#### Topic Model

A topic model is used to find the probability distribution of hidden topics in text. The Latent Dirichlet Allocation (LDA) model is the most popular topic model. Martinez-Torres et al. (2015) applied the LDA model to obtain Starbucks consumers’ preferences. Liu et al. (2018) used the LDA model to discuss the main topics of an online health community. Although using the LDA model to analyze online community text has gradually attracted scholars’ attention, there is little research on the online technology community from a gender perspective. The difference of information topics is one of the bases for some scholars to judge the gender differences (Salem et al., 1997). Therefore, from the perspective of gender, this paper uses the LDA model to divide the topic categories under different genders.

#### Sentiment Analysis

Sentiment analysis refers to the detection and classification of the sentiments expressed by opinion holders (Kim et al., 2017). The purpose is to judge the positive, negative, and neutral meaning of the text, making online-generated content easier to process and understand (Thelwall et al., 2010). Sentiment analysis based on machine learning is a common method. It is a supervised machine learning method, which is scientific and efficient, and does not require excessive human involvement. Pang et al. (2002) first applied machine learning to sentiment analysis of film reviews. Tripathy et al. (2016) used machine learning methods to divide Weibo and online comments into positive, neutral, and negative texts. In recent years, scholars applied sentiment analysis to gender difference research. Zhang et al. (2013) found that women are more likely to express positive and negative emotions in online forum communication than men.

Existing research mainly focuses on sentiment recognition, without further consideration of the impact of shared information on user activity (Chung and Zeng, 2020). The research of this content not only is beneficial to the application field of text mining methods but also can realize the practical value of text mining methods more effectively. Therefore, this paper will use sentiment analysis to determine the sentimental tendencies of different-gender users and analyze the impact of sentimental tendencies of different-gender users on user activity under each topic.

## Materials and Methods

### Choice of Target Community

The Python community is one of the most active online technology communities in China. It is a non-profit online technology exchange that aims to help users to learn technology, share learning experiences, and make friends with like-minded technology learners. In addition, considering that the Python community is not gender specific, this paper chooses the post text of the Python community to explore the information behavior of men and women.

### Ethical Considerations

The study was approved by Python community managers. Before the study began, we fully explained the study purpose and methods to the community managers, to ensure that personal information would safe and protected and to ensure that the community managers could withdraw from the study at any time without damage. After informed consent of community managers, all data were properly obtained.

### Measures

We designed a multi-threaded crawler code, collected all posts of the Python community from January 2006 to April 2018, and obtained a total of 302,914 posts. Through data cleaning and sorting, in the end, a total of 225,875 valid posts about the Python community were obtained, of which 172,487 were from male users and 53,388 were from female users.

### Data Analysis

Latent Dirichlet Allocation is a three-layer Bayesian theme model proposed by Blei in 2003. The unsupervised learning method is used to discover the subject information implicit in text. The purpose is to have no guidance of learning from the implied semantic dimension found in the text, namely “topic” or “concept.” The essence of the LDA topic model is to discover the topic structure of the text by using the co-occurrence feature of the word in the text. This method does not require any background knowledge about the text. The implicit semantic representation of the text can be used to model the linguistic phenomena of “one word polysemy” and “one meaning polysemy,” which makes the search results obtained by the search engine system match the users’ query on the semantic level, rather than just the intersection on the lexical level (Lu Y. J. et al., 2013). This model can effectively extract the hidden topics of documents and cluster them. Because of the superiority of the LDA model, it has become a common method to find large-scale text topics. In this study, documents are posts made by users in the community. We use the LDA model to analyze gender differences in different topics, use sentiment analysis to determine the sentiments of all users’ posts, and use multiple regression analysis to analyze whether different emotional tendencies under different topics affect user activity. The proportion of positive and negative emotions of male users and female users under each topic is taken as an independent variable, and the number of posts of male users and female users under each topic is taken as a dependent variable.

## Results

### Topic Model Results

First, we performed data preprocessing on the post text. In this method, we first split the complaints into words using Jieba, a Chinese Natural Language Processing (NLP) tool. Meanwhile, we also filtered the stop words using a Chinese stop words list, which, in total, includes 1,893 words. This Chinese linguistic list is often used in Chinese natural language processing. After that, we used the LDA model for analysis. To overcome this limitation of LDA, the Gibbs sampling algorithm was used to estimate model parameters. The parameter estimation method of Heinrich (2005) was used to set parameter α = 50/K, parameter β = 0.1. By adjusting related parameters, and conducting multiple experiments and comparisons, this paper finds that when the number of topics is *K* = 4, and the number of keywords is 10, the similarity of each topic is small, and the topics are well clustered. The words of each topic are most easily summarized, and the probability of each topic word is high, which indicates that the topic classification effect is the best at this time.

The topic information of male and female users is shown in Tables 1, 2. The naming of each topic is mainly based on the following two steps: Firstly, group the subject words with similar meaning into a group, and name the phrase. For example, female users in Table 2 have three main keywords in topic 1: seeking (qq, com, and thank you), information (data, program, code, and indentation), and help (newbie, learning, and landlord). The second step is to name the topic based on the relevance between the phrases under each topic. Thus, topic 1 of the female users is named “seeking information help.” It can be seen that the topics of different-gender users are the same; that is, the four topics are seeking information help, providing information help, technology exchange, and making friends and advertising. In terms of the proportion of posts on each topic, male and female users mostly participate in two kinds of topics: seeking information help and providing information help. In topic 1, men and women used the same words: thanks, landlord, and qq, indicating that both male and female users would prefer to obtain information from the landlord through qq communication software. In topic 2, men and women used the same words: course, share, simple, feel, and basis, indicating that male and female users will share some simple or basic learning tutorials in the community. In topic 3, men and women used the same words: import, function, run, and support, indicating that men and women users will discuss specific technical communication issues such as code writing and arithmetic functions in the community. In topic 4, men and women used the same words: reply, Baidu, and make friends, which shows that male and female users in the community want to make friends through a Baidu Tieba account, which will help long-term academic communication and interaction in the future.

**TABLE 1:** Topic information for male users.

	**Topic 1**	**Topic 2**	**Topic 3**	**Topic 4**
Subject words	Thanks	Learn	Code	Reply
	Landlord	Novice	Import	Baidu
	Lz	Course	Function	Language
	Thank you	Share	Version	Make friends
	qq	Master	Run	Come
	Gratitude	Simple	File	Speak freely
	Trouble	Recommend	Method	Hello
	Thanks very much	Range	Seemingly	Same request
	Explanation	Feel	Program	Understand
	Com	Basis	Support	Bin
Topic name	Seeking information help	Providing information help	Technology exchange	Making friends and advertising
Number and proportion of posts	66,767 (38.7%)	58,084 (33.6%)	25,293 (14.6%)	22,343 (12.9%)

**TABLE 2:** Topic information for female users.

	**Topic 1**	**Topic 2**	**Topic 3**	**Topic 4**
Subject words	Code	File	Import	Reply
	Landlord	Course	Run	Okami
	Learn	Simple	Function	Seemingly
	Thanks	Version	Support	Solve
	Indent	Material	Input	Install
	Procedure	Feel	Module	Make friends
	Novice	Program	Download	Baidu
	Com	Share	Linux	Gratitude
	qq	Reading	Def	Please
	Data	Basis	Language	Newcomer
Topic name	Seeking information help	Providing information help	Technology exchange	Making friends and advertising
Number and proportion of Posts	20,175 (37.8%)	13,861 (26.0%)	5,740 (10.8%)	13,612 (25.5%)

### Sentiment Analysis Results

In this paper, based on the Python language, sentiment analysis is performed on the effective posts of male users and female users under different topics. The distribution of sentiment tendency between male and female users under each topic is shown in Table 3. It can be seen that under the topic of seeking information help, the proportion of male users expressing positive emotion is 38%, and the proportion of female users expressing positive emotion is 59%, while male users relatively show more negative emotion (25%). Under the topic of providing information help, the proportion of female users expressing positive emotion (69%) was significantly higher than that of male users (44%). Under the topic of making friends and advertising, the proportion of female users expressing positive emotions (66%) is also higher than that of male users (55%). Under the topic of technology exchange, the distribution of emotional tendency of male users and female users is not significantly different.

**TABLE 3:** Male and female sentiment distribution results.

	**Sex**	**2 (Positive)**	**1 (Neutral)**	**0 (Negative)**
Topic 1: seeking information help	Male	25,479 (38%)	24,821 (37%)	16,467 (25%)
	Female	11,849 (59%)	7,512 (37%)	814 (4%)
Topic 2: providing information help	Male	25,556 (44%)	12,315 (21%)	20,213 (35%)
	Female	9,546 (69%)	2,964 (21%)	1,351 (10%)
Topic 3: technology exchange	Male	10,858 (43%)	6,646 (26%)	7,789 (31%)
	Female	2,659 (46%)	1,120 (20%)	1,961 (34%)
Topic 4: making friends and advertising	Male	12,331 (55%)	3,744 (17%)	6,268 (28%)
	Female	8,928 (66%)	3,332 (24%)	1,352 (10%)

Furthermore, a chi-square test was carried out for the four different topics to study the relationship between male and female users and emotion. The results are shown in Table 4. In those cases where the *p*-value is lower than 0.05, the null hypothesis can be rejected, so it can be determined that there are significant differences between gender and emotion under the four topics.

**TABLE 4:** Chi-square test results on different topics.

	**Chi-square tests**	
	**Chi-sq.**	***p***
Topic 1: seeking information help	4,843.947	0.000
Topic 2: providing information help	3,762.28	0.000
Topic 3: technology exchange	114.806	0.000
Topic 4: making friends and advertising	1,721.606	0.000

Table 5 details the results of the 10-fold cross-validation classifier for male and female users, with accuracy and recall rates above 70%, which means the method has good accuracy.

**TABLE 5:** Results of the classifier for male and female users.

		**Topic 1**			**Topic 2**			**Topic 3**			**Topic 4**		
		**2**	**1**	**0**	**2**	**1**	**0**	**2**	**1**	**0**	**2**	**1**	**0**
Accuracy	Male	0.801	0.831	0.776	0.729	0.841	0.777	0.816	0.793	0.788	0.822	0.735	0.857
	Female	0.791	0.858	0.876	0.785	0.852	0.797	0.837	0.773	0.795	0.862	0.772	0.863
Recall	Male	0.798	0.815	0.807	0.765	0.735	0.727	0.809	0.795	0.817	0.825	0.711	0.842
	Female	0.767	0.847	0.838	0.732	0.835	0.729	0.851	0.761	0.801	0.854	0.731	0.817

**2 means positive emotion, 1 means neutral emotion, and 0 means negative emotion*.*

### Regression Analysis Results

After topic-oriented sentiment analysis, we want to explore the differential influence of gender in emotional tendency on user activity, that is, to analyze the regulatory effect of gender in emotional tendency on user activity. Therefore, this paper establishes a group regression model to further analyze the relationship between the activity of male users and female users under each topic and their emotional tendencies. At the same time, a seemingly unrelated regression test (SUEST) is performed on the differences in regression coefficients between groups. The analysis results are shown in Table 6. Under topic 1, from the tests of model 1 and model 2, and the corresponding inter-group coefficient difference test, it can be seen that the negative emotion of male and female users is negatively correlated with user activity, and the positive emotion of male and female users is positively correlated with user activity. The inter-group SUEST test of the two sets of regression coefficients of negative emotions was significant at the 1% level, indicating that the effect of a user’s negative emotions on activity was significantly different in each gender. Moreover, the coefficient value for women is greater than the coefficient value for men (2.525 > 0.974), indicating that female users are more susceptible to negative emotions than male users. The inter-group SUEST test of the two sets of regression coefficients of positive emotions was significant at the 1% level, indicating that the effect of a user’s positive emotions on activity was significantly different in each gender. Moreover, the coefficient value of women was greater than the coefficient value of men (1.325 > 1.291), indicating that female users are more susceptible to positive emotions than male users. Under topic 2, model 3, model 4, and the corresponding inter-group coefficient difference test show that the influence of male users’ emotion on activity is not significant, while that of female users is significant. The influence of negative emotion and positive emotion on activity of users has significant differences in each gender. Further comparison of regression coefficients shows that female users’ activeness is more susceptible to the influence of negative and positive emotions than male users. Under topic 3, from model 5, model 6, and the corresponding inter-group coefficient difference test, it can be seen that the negative emotion of male and female users has no significant impact on user activity, and the positive emotion of male and female users has a significant positive correlation with user activity. There are significant differences in the influence of users’ negative and positive emotions on the activity between genders. Further comparison of regression coefficients shows that female users’ activeness is more susceptible to the influence of negative and positive emotions than male users. Under topic 4, from the tests of model 7 and model 8, and the corresponding inter-group coefficient difference test, it can be seen that the negative emotions of male and female users are significantly negatively correlated with user activity, while the positive emotions of male and female users have no significant impact on user activity. There are significant differences in the influence of users’ negative and positive emotions on the activity between genders. Further comparing the regression coefficient, we can see that compared with male users, female users’ activeness is more susceptible to the influence of negative and positive emotions. Overall, compared with male users, female users’ activity is more susceptible to emotional tendencies.

**TABLE 6:** Regression results of emotional tendencies and user activity.

**Variables**	**Dependent (user activity)**											
	**Topic 1**		**SUEST**	**Topic 2**		**SUEST**	**Topic 3**		**SUEST**	**Topic 4**		**SUEST**
	**Female**	**Male**		**Female**	**Male**		**Female**	**Male**		**Female**	**Male**	
								
	**Model 1**	**Model 2**		**Model 3**	**Model 4**		**Model 5**	**Model 6**		**Model 7**	**Model 8**	
Negative emotions	−2.515**	0.974***	Chi-sq. = 11.89 *P* = 0.0006	−6.559**	−1.398	Chi-sq. = 6.02 *P* = 0.014	−1.336	−1.163	Chi-sq. = 4.94 *P* = 0.0263	−2.837*	−1.161*	Chi-sq. = 7.53 *P* = 0.0061
Positive emotions	1.325**	1.291*	Chi-sq. = 7.67 *P* = 0.006	1.234**	1.037	Chi-sq. = 4.89 *P* = 0.027	1.246**	1.236*	Chi-sq. = 5.81 *P* = 0.016	0.581	0.314	Chi-sq. = 6.79 *P* = 0.0092
Constant	−446.707	27.768		66.548	107.952		36.857	20.875		361.808	33.399	
*R*^2^	0.998	1.000		0.926	0.990		0.998	0.999		0.964	0.981	
Adj – *R*^2^	0.997	1.000		0.889	0.985		0.997	0.998		0.946	0.957	
*F*	916.124	1,900.342		25.135	197.406		1,226.481	1,909.681		53.903	67.134	

*p < 0.05, **p < 0.01, ***p < 0.001. “SUEST” means “seemingly unrelated regression test”.

## Discussion

The purpose of this study was to broaden the understanding of online technology communities by comparing the differences between male and female users in topics, emotions, and the impact of emotions on activity. This paper discusses the different topics of male and female users through the LDA model. Then, the sentiment analysis method is used to compare the emotional tendency of male and female users. Finally, multiple linear regression analysis is used to discuss the different influences of user emotions on activity under different topics. The results show the following: (1) Online technology community posts are divided into four topics: seeking information help, providing information help, technical exchange, and making friends and advertising. Male users tend to provide information help, while female users prefer to participate in the topic of making friends and advertising. (2) When communicating in the technology community, most of the male and female users express positive emotions, but the frequency of female users expressing positive emotions is higher. (3) The emotion of male and female users under different topics in the community has a significant impact on their activity, but the impact is different. In comparison, the activity of female users is more susceptible to emotional tendencies.

### Gender Difference in Python Community Topic Classification

Through the analysis of the LDA model, we found that the topics of male and female users in the Python community are the same, which shows that the goals and ideas of male and female users participating in the community are generally consistent. In addition, we found that male and female users are most involved in two kinds of topics: seeking information help and providing information help. The reason is that as a computer language, Python has a high technical threshold, so most users in the Python community are beginners. Their needs in the community are mainly some introductory video tutorials, learning materials, and complete code. Barcellini et al. (2008) also pointed out that most Python community users tend to provide references to usage and personal experience, code, and examples, which is supported by our findings.

A surprising finding of this study is that the distribution of posts of male users and female users under different topics also presents certain differences. Under the topic of providing information help, male users in the technology community will be more proactive in providing information help to other users. This finding differs from research by Thelwall et al. (2010), which found that women in online social networks often play the role of contributors. Potential explanations are as follows: different social backgrounds make gender differences in online communities unlikely to produce uniform results (Spears and Lea, 1994). Socio-cultural theory points out that social culture will affect gender differences (Wood and Eagly, 2012). The specific social background of the online technology community will lead to different gender differences. In this particular social background, the online technology community is more about technology knowledge exchange. Women pay more attention to interpersonal communication (Shaw and Gant, 2002); men are willing to show self-confidence, which is an inherent characteristic of boys (Croson and Gneezy, 2009); and positive stereotypes make men more active in providing information to show self-confidence in the online technology community. This paper confirms the results of Ginossar (2008), which show that men are more inclined to publish documents providing information. This is also consistent with the selectivity hypothesis, which holds that men’s processing information is not as thorough as women’s. Therefore, women read their search results more thoroughly and in detail, with no advantage over men’s knowledge obtained by quickly scanning their more relevant search results. Under the topic of making friends and advertising, female users are more willing to make friends with other users in the technical community and publish advertisements for their own Python classes. This finding is consistent with the findings of Shaw and Gant (2002), who found that women prefer to use the Internet for interpersonal communication. Beck et al. (2012) also reported similar findings from the perspective of trust theory. They observed that women are more likely to be trusted than men because women are more inclined to social relationships. It can be seen that the research in this paper further supports this conclusion from the perspective of the online technology community.

### Gender Differences in Sentiment Analysis

Sentiment analysis shows that users of different genders express more positive emotions in the technology community. In contrast, female users convey positive emotions more frequently, while male users convey negative emotions more frequently. The gender belief system model states that people’s perceptions of men and women are constrained by social expectations (Deaux and Kite, 1987). Belief systems include gender stereotypes, attitudes to roles that are appropriate for each gender, and perceptions of those who violate these expectations (Deaux and Lewis, 1984; Deaux et al., 1985; Berndt and Heller, 1986). Therefore, the female characteristics of stereotypes are also expected to have female gender roles. That is, roles, traits, and appearances form a coherent, interconnected system: Women often have characteristics that are related to emotional expression (that is, enthusiasm, kindness, and attention to others) (Spence and Helmreich, 1978). As a result, women show more positive emotions.

Specifically, under the topic of seeking information help, although most users express positive and neutral emotions, male users relatively show more negative emotions, which shows that male users are more likely to express impatience and dissatisfaction in the process of technical learning. A possible explanation for this finding is that with negative emotions, male and female users treat information differently. In contrast, men are more likely to be emotionally depressed (Matud, 2004). Men use distracting strategies to repair sadness and ignore the problems to be solved. However, women take a positive attitude and use more detailed methods to solve the problem (Martin, 2003). Women often seek social support as a coping mechanism and use positive emotions to cope with the negative emotions brought about by events (Day and Livingstone, 2003; Matud, 2004). The selective hypothesis also validates this view from the side. The selective hypothesis indicates that as comprehensive processors, women tend to use comprehensive strategies to comprehensively process information and try to absorb all available clues. Although capacity constraints in activities may prevent women from achieving this goal, they usually try to conduct a comprehensive and detailed analysis of all available information (Meyers-Levy, 1989; Meyers-Levy and Maheswaran, 1991; Meyers-Levy and Sternthal, 1991). Consistent with the socio-cultural perspective, it points out that compared with men, women are socialized to decode emotions, women show higher sensitivity and responsiveness, and women are more able to regulate their dissatisfaction (Meyers-Levy and Loken, 2015). As a result, men are more likely to show irritability and dissatisfaction (McRae et al., 2008). Under the topics of providing information help and making friends and advertising, female users actively help and socialize in the technology community. This finding can be explained by females’ common values, which emphasize the maintenance of social and interpersonal harmony (Kurt et al., 2011). Under the topic of technology exchange, both male users and female users expressed more positive emotions with little difference, reflecting a good knowledge communication atmosphere in the Python community. With the increasing distribution and globalization of open source software, people can discuss and exchange at any time and at any stage, which is beneficial to the value of the online technology community (Gasser et al., 2003).

### Effects of Gender Differences in Sentiment on User Activity

Through multiple linear regression analysis, we also found that the influence of user sentiment on user activity is different under different topics. Generally speaking, positive sentiment can promote the user’s activity under each topic, while negative sentiment can inhibit it. In comparison to male users, the activity of female users is more susceptible to sentiment. This supports the gender role theory, which states that the assessment of events varies by gender (Tamres et al., 2002; Sarrasin et al., 2014). That is, gender roles can be used by men and women to self-regulate their behavior. The emotions experienced by men and women can serve as feedback and reinforce behavioral change in more gender-typical ways. Women are more likely to assess an event as stressful than men. In addition, gender role theory also shows that men are socialized as independent and depressing emotions, while women are socialized as enthusiastic, supportive, compassionate, and sensitive to the feelings of others (Reevy and Maslach, 2001). Women may be particularly sensitive to environmental cues and emotions due to their common gender tendencies, which makes them more likely than men to change their behavior in a way that is appropriate for the environment (Wood and Eagly, 2012). As a result, women are more susceptible to emotions in their activities.

Specifically, under seeking information help, the activity of male and female users is positively correlated with their positive emotions and negatively correlated with their negative emotions. This indicates that positive emotions promote the technical learning enthusiasm of technical community users, while negative emotions will inhibit their knowledge-seeking behavior. At the same time, we found that negative emotions have more influence on female users than male users. The potential explanation for this finding may be that, as women are more cautious, they show greater sensitivity and responsiveness to stimuli that may have a negative impact than men (Meyers-Levy and Loken, 2015). This is also found in the marketing environment. Compared with men, women are more likely to have negative views on negative information (Putrevu, 2010).

Under the topic of providing information help, the correlation between male user activity and their emotional tendency is not significant, while female users are just the opposite. Their activity is significantly positively correlated with positive emotion and negatively correlated with negative emotion, and the absolute value of the positive emotion correlation coefficient is greater than negative emotion; this result also shows that male users are more rational when providing information help to others and are not affected by emotional tendency, while female users are more emotional, and negative emotions will hinder their information support behavior. This again supports Meyers-Levy and Loken (2015) view that because women are more cautious, they are more sensitive and responsive to stimuli that may have a negative impact than men. The same findings have been found for online medical communities. There are significant gender differences in comparing information provided by male and female users. Women will give priority to emotional issues, while men mainly focus on actual tasks and information-related exchanges (Mo et al., 2009).

Under the topic of technology exchange, the activity of male and female users is positively correlated with their positive emotions, while the negative emotions are not significant. This result shows that users in the community are mainly driven by their motivation of information demand. When exchanging knowledge, positive emotions will make them willing to communicate on technical issues, while negative emotions will not significantly affect technology exchange activities. This can be explained by the theory of immersion. People are interested in an activity and a transaction and fully participate in it. After the experience of enjoyment is formed, users will maintain the experience through repeated behaviors, that is, immersion experience (Chen et al., 1999). The human–computer interaction in the network provides users with a means of immersive experience; the knowledge sharing and communication behaviors of online communities contribute to the emergence of immersive experience (Yan et al., 2013). Immersive experience is a positive emotional experience that promotes continuous technical communication behavior among users (Lin et al., 2005). Therefore, under the topic of technology exchange, increasing the positive emotions of users can improve users’ activity in the technology community.

Under the topic of making friends and advertising, positive emotions do not affect making friends and advertising behavior, but negative emotions can significantly inhibit the social and promotional behavior of male and female users in the community. As Matsumoto (1996) said, personal negative emotions can affect the harmony of the whole social group. From social support theory, making friends and advertising belong to companionship support in social support, and thus will extend the active time of users in the forum (Wang et al., 2017). It can be seen that tracking users’ emotional tendencies in time is conducive to the long-term development of the community.

### Contributions and Limitations

The contributions of this paper are as follows: firstly, this paper studies the gender differences of users in an online technology community starting from the topic classification of the content of the posts, which makes up for the lack of understanding of gender differences in online technology communities in previous studies. Secondly, different from the commonly used questionnaires and field interviews, this paper uses a large number of post texts as data sources, using the LDA topic model, machine learning, and other methods for topic classification and correlation analysis, providing new data sources and research approaches for relevant research on gender differences. Finally, the empirical results of emotion and user activity under different topics should reveal the aggregation behavior of users under different topics in the community and enrich the research field of emotion analysis.

Our research results have much practical significance, which can promote the interaction of community users and the construction of an online technology community information system. In our research on male and female users, these differences help to better understand their needs and clarify how to better serve these users. First of all, we use the LDA model to identify online technology community topics, which is beneficial for community managers to guide different-gender users. An online technology community information system can provide male and female users with recommendations and requests to reply to posts with different topics. In the aspect of ergonomics realization, it is beneficial for both respondents and questioners to provide information topics of interest to target users. Secondly, through sentiment analysis of community posts, it provides the basis for users to provide refined management. In the process of technical communication, community managers and users can better interact with each other and maintain a positive attitude in a more effective and personalized way; it can help community managers to divide community users into groups and help the community realize the refined management of users. Thirdly, the research results provide important management implications for community development. Through regression analysis, this paper studies the relationship between sentimental tendency and user activity of different-gender users under different topics, which is beneficial for community managers to monitor and adjust user activity under different topics of the community through the change of sentimental tendency, so as to realize the sustainable development of the community. Finally, this study provides strategies for negative sentiment management. Community managers should pay more attention to the discussion of female users to alleviate the spread of negative sentiments. These designs will help optimize human–computer interaction and improve the service of the online technology community.

Due to the limitations of conditions and technologies, this paper only crawled the text data in the Python community. Although the size of the data is large, there are still some limitations in the selection of the technical community. In future research, the author will consider obtaining the text data from other online technology communities and analyze related contents to further test the research methods and results of this paper. At the same time, in the future, the method of time series analysis can be considered to study the changes in the activity and emotion of users of different genders under various topics and then explore the rules of user role evolution, so as to realize the dynamic expansion of this study.

## Data Availability Statement

The datasets generated for this study are available on request to the corresponding author.

## Author Contributions

BS, HM, and CY contributed the conception and design of the study. CY organized the database. HM performed the statistical analysis and wrote the first draft of the manuscript. BS, HM, and CY wrote sections of the manuscript. All authors contributed to manuscript revision and read and approved the submitted version.

## Conflict of Interest

The authors declare that the research was conducted in the absence of any commercial or financial relationships that could be construed as a potential conflict of interest.
